# Chlorpyrifos Accumulation Patterns for Child-Accessible Surfaces and Objects and Urinary Metabolite Excretion by Children for 2 Weeks after Crack-and-Crevice Application

**DOI:** 10.1289/ehp.6984

**Published:** 2004-09-23

**Authors:** Paromita Hore, Mark Robson, Natalie Freeman, Jim Zhang, Daniel Wartenberg, Halûk Özkaynak, Nicolle Tulve, Linda Sheldon, Larry Needham, Dana Barr, Paul J. Lioy

**Affiliations:** ^1^Environmental and Occupational Health Sciences Institute, Exposure Measurement and Assessment Division, Rutgers University and the University of Medicine and Dentistry of New Jersey, Robert Wood Johnson Medical School, Piscataway, New Jersey, USA; ^2^National Exposure Research Laboratory, U.S. Environmental Protection Agency, Research Triangle Park, North Carolina, USA; ^3^Contemporary Pesticide Laboratory, Centers for Disease Control and Prevention, Atlanta, Georgia, USA

**Keywords:** biomarker, child, children, chlorpyrifos, crack-and-crevice, indoor chemical use, pesticide

## Abstract

The Children’s Post-Pesticide Application Exposure Study (CPPAES) was conducted to look at the distribution of chlorpyrifos within a home environment for 2 weeks after a routine professional crack-and-crevice application and to determine the amount of the chlorpyrifos that is absorbed by a child living within the home. Ten residential homes with a 2- to 5-year-old child in each were selected for study, and the homes were treated with chlorpyrifos. Pesticide measurements were made from the indoor air, indoor surfaces, and plush toys. In addition, periodic morning urine samples were collected from each of the children throughout the 2-week period. We analyzed the urine samples for 3,5,6-trichloropyridinol, the primary urinary metabolite of chlorpyrifos, and used the results to estimate the children’s absorbed dose. Average chlorpyrifos levels in the indoor air and surfaces were 26 (pretreatment)/120 (posttreatment) ng/m^3^ and 0.48 (pretreatment)/2.8 (posttreatment) ng/cm^2^, respectively, reaching peak levels between days 0 and 2; subsequently, concentrations decreased throughout the 2-week period. Chlorpyrifos in/on the plush toys ranged from 7.3 to 1,949 ng/toy postapplication, with concentrations increasing throughout the 2-week period, demonstrating a cumulative adsorption/absorption process indoors. The daily amount of chlorpyrifos estimated to be absorbed by the CPPAES children postapplication ranged from 0.04 to 4.8 μg/kg/day. During the 2 weeks after the crack-and-crevice application, there was no significant increase in the amount of chlorpyrifos absorbed by the CPPAES children.

Eighty percent of U.S. households use pesticides more than once a year in and around their homes (Davis et al. 1992; Whitmore et al. 1994). Many of the pesticides applied indoors are semivolatile, with vapor pressures ranging from 10^−2^ to 10^−8^ mm Hg (Dalaker and Naifeh 1997). Once applied indoors, semivolatile pesticides can vaporize from treated surfaces and can distribute in and on targeted and nontargeted surfaces and objects (Byrne et al. 1998; Gurunathan et al. 1998; Lewis et al. 2001; Wright et al. 1984). This raises concern about exposures because U.S. householders, including children, can spend up to 90% of their time indoors within or around treated areas (Savage et al. 1981). Children in pesticide-treated homes may be exposed to pesticides via multiple routes and from multiple media. Given their inherent biologic vulnerabilities and characteristic behaviors that are different from those of adults, children can be particularly susceptible to the effects of pesticides (Aprea et al. 2000; Bearer 1995; Freeman et al. 1997; Guzelian et al. 1992; Reed et al. 1999).

In 1996, the Food Quality Protection Act (FQPA) mandated that contributions from all routes of exposure and from all possible sources be considered when setting food tolerance levels for pesticides, paying particular attention to address the potential risks to infants and small children (FQPA 1996). Several studies have used direct and indirect measures to try to estimate the total pesticide uptake by children via the inhalation, dermal, and nondietary ingestion routes after an indoor pesticide application (Byrne et al. 1998; Gurunathan et al. 1998; Lewis et al. 2001). Pesticide body burden levels estimated from environmental concentrations have been reported after either broadcast (Gurunathan et al. 1998) or homeowner/professional crack-and-crevice applications (Byrne et al. 1998; Lewis et al. 2001). No studies thus far have serially collected biomarker samples from children residing within treated homes to allow a comparison between body burden estimated from environmental data and body burden estimated from biomarker levels. Given that information regarding pesticide uptake by children in treated homes is needed to assess the health risks for exposed children, the lack of information on the time course of body burden levels after professional indoor application is a gap in the currently available research.

A detailed multimedia/multipathway 10-home residential study, referred to as the Children’s Post-Pesticide Application Exposure Study (CPPAES), was conducted to provide information on the release and movement of chlorpyrifos, a semivolatile pesticide (vapor pressure, 1.87 × 10^−5^ mm Hg at 20°C), within a residential environment and within children living in this environment over time after an application. The scientific approach involved collecting environmental samples from a treated home coupled with biomarker samples from a child living in the treated home, for 2 weeks after a routine crack-and-crevice application of chlorpyrifos. CPPAES was designed to evaluate the extent of aggregate chlorpyrifos exposure for children living within treated homes. The general concept for this study was outlined during a workshop held by the International Life Sciences Institute (ILSI 2000). The study was carried out between 1999 and 2001, before the U.S. Environmental Protection Agency (EPA) phased out indoor residential use of organophosphate pesticide.

## Materials and Methods

### Study design.

Ten residential homes (identified as H1–H10) were selected for CPPAES based on the criteria that they applied pesticides on a routine basis and had a child between 2 and 5 years of age who spent most of his or her time indoors at home. Each of the CPPAES homes was located in urban areas within New Jersey. The homes varied in size (34–96 m^2^) and style. For the protection of human subjects, the study design was thoroughly evaluated and approved by the institutional review board committee of the University of Medicine and Dentistry of New Jersey and the U.S. EPA.

### Pesticide application.

The commercial product Dursban 2.E. or Dursban L.O. containing the insecticide chlorpyrifos [*O*,*O*-diethyl-*O*-(2-isopropyl-6-methyl-4-pyrimidinyl) phosphorothioate, CAS No. 2921-88-2] was applied to each of the CPPAES homes reportedly as a 0.25–0.5% water emulsion. Until recently and throughout the study period, chlorpyrifos was one of the most commonly used household insecticides within the United States used by homeowners, renters, and professional applicators to control cockroaches, fleas, and termites (U.S. EPA 2000). A licensed pesticide applicator applied the pesticide solution to each of the homes via a crack-and-crevice mode of application. The applications were made using a hand-pump compressed air sprayer (tank capacity, 1 gallon) with a pin stream nozzle, spraying with a downward-directed nozzle tip 12–16 inches from the floor. Applications were made to the cracks and crevices of the homes and in some cases along the perimeters of the walls behind appliances or furniture. The applications lasted approximately 15 min per home as the applicator examined each home for cracks and crevices and evidence of roach trails. Approximately 60–700 mL of the chlorpyrifos solution was reportedly sprayed in each CPPAES home. A sample of the pesticide solution applied within each home (except H1) was collected from the pesticide applicator, and the samples were analyzed in the laboratory. The amount of pesticide applied in each home was then based on the estimated volume of the pesticide solution applied. Although the study was designed to make uniform applications in each home, the analytical results indicated that the amount of chlorpyrifos applied within homes H8–H10 (4.1 × 10^−7^ to 4.3 × 10^−6^ g) was considerably lower than what was applied in homes H2–H7 (0.07–0.6 g). A sample of the pesticide application solution was not available for H1; however, based on the chlorpyrifos levels measured in the indoor air postapplication, the applied amount in H1 was probably similar to amounts applied in homes H2–H7.

In homes H3, H5, and H8, the pesticide was applied in all rooms. For homes H1, H2, and H4, the pesticide was applied in all the rooms except the bathrooms. For homes H6, H7, and H9, it was not applied in the parents’ bedrooms; for H10, pesticide was not applied in two of the bedrooms. During the crack-and-crevice application, the study participants left the treated homes and no sampling was conducted. After the application, re-entry did not occur for 3 hr. An exception was H10, where during this time the participants restricted their movements to the untreated portions of the house rather than vacating the home. The windows in all of the homes were “cracked” open during this 3-hr period.

### Sampling scheme.

A 2-week multimedia sampling effort was carried out before and after an indoor crack-and-crevice application. Environmental samples were collected over time for measuring chlorpyrifos in the indoor environment. Simultaneously, biomarker samples were obtained from the participating children living within the treated homes. Preapplication measurements were made from the CPPAES homes on the day before the day of pesticide application. A crack-and-crevice pesticide application was then made to each of the CPPAES homes on what is designated day 0. Postapplication measurements were made on days 1, 2, 3, 5, 7, 9, and 11 after the day of application. The sampling scheme is presented in Table 1.

### Samples collected.

In each CPPAES home, measurements were taken in two rooms that had been treated with the pesticide: either in the child’s main play area, designated “A,” and/or in or near the child’s bedroom area, “B.”

Time-weighted average measurements for chlorpyrifos vapor and aerosol were obtained in chlorpyrifos-treated rooms (H1–H9 samples collected in area A; H10 samples collected in area B). The indoor air samples were collected using a low-flow pump with a PM_10_ (particulate matter ≤10 μg) inlet and a carbon-impregnated filter. Collection and extraction methods for the air samples were developed by Gurunathan et al. (1998). The sampling time per sample spanned the time interval between each visit (i.e., days –1 to 0, 0–1, 1–2, 2–3, 3–5, 5–7, 7–9, and 9–11). Postsampling, the filters were extracted in 10 mL toluene via sonication and concentrated down to a sample volume of 5 mL. Mean recoveries for chlorpyrifos from laboratory controls were 101% [coefficient of variation (CV), 7.8%].

Surface wipe samples were collected within treated rooms on days –1, 1, 2, 3, 5, 7, 9, and/or 11 from nontargeted surfaces (i.e., areas not directly sprayed with the pesticide). The Lioy-Weisel-Wainman (LWW) sampling method as described by Gurunathan et al. (1998), and the method of Lioy et al. (2000) was used to collect the pesticide wipe samples by the movement of a C18-impregnated Teflon filter (moistened with isopropanol) within a 100 cm^2^ template.

The LWW sampler was used to collect wipe samples from smooth surfaces in both areas A and B. The wipe samples obtained from area A were collected from floor surfaces. All of the wipe samples obtained from area B, except for H1, H2, and H8 (day –1), were also collected from floor surfaces. Two-week area B samples collected from homes H1 and H2 were obtained from a dresser, 0.1–0.8 m above the floor. The H8 day –1 area B sample was collected from a windowsill, 0.6 m above the floor. Because of limited resources, except for in homes H2, H9, and H10, samples from area B were not collected on days 5 and 9. No LWW samples were collected from area B in H8 as the floor was carpeted. Each LWW wipe sample was collected from a different location within each home to prevent the surface activation previously noted by Gurunathan et al. (1998). However, whenever possible, the sampled areas were adjacent to the previous samples. Postsampling, the LWW filters were extracted in 5 mL of isopropanol via sonication. Mean recoveries for chlorpyrifos from laboratory controls were 106% (CV, 4.9%). The data were used to estimate the amount of chlorpyrifos distributed on open surfaces in the treated home environment.

Chlorpyrifos measurements were also made on samples collected from indicator toys placed within CPPAES treated rooms (H1–H3, H5–H9 samples collected in area A; H4 and H10 samples collected in area B). Toys were placed indoors immediately after the 3-hr re-entry period, and each was sequentially removed for chlorpyrifos analysis on days 1, 2, 3, 5, 7, 9, and 11 postapplication. A duplicate toy was collected on days 2, 5, and 9 postapplication from homes H3–H10. For H2, a duplicate sample was available only for day 2. For H5, a duplicate toy was collected on days 2, 5, 9, and 11 postapplication. “Sweet Stuffs” toys from the “The First Years Collection” purchased at Toys R Us (Watchung, NJ) (surface areas ~ 125–150 cm^2^) were used as the indicator toys. They were placed in a birdcage to limit the children’s interactions with the toys but at the same time not sheltering the toys from the movement of pesticide in the air. Plush toys were used because they are a potential sink for pesticides accessible to children within residential homes. Moreover, plush toys can serve as a surrogate for any sorbant medium present indoors with polyfoam filler, such as furniture upholstery and bedding.

A combination of toy surface wipes and toy extractions were analyzed from the duplicate toys to evaluate both dislodgeable and total components of the pesticide in/on the duplicate toys. A surface wipe of the duplicate toys was collected before the full extraction of the toys. The surface of each plush toy was wiped using isopropanol-impregnated swabs. The swab wipes were then extracted in 10 mL of isopropanol via sonication and concentrated down to a sample volume of 5 mL. Mean recoveries for chlorpyrifos from laboratory controls were 102% (CV, 3.2%). The plush toys were then extracted in 200 mL of hexane via sonication and concentrated down to a sample volume of 5 mL (Gurunathan et al. 1998). Mean recoveries for chlorpyrifos from laboratory controls were 96% (CV, 6.2%).

To estimate chlorpyrifos bioaccumulation in the CPPAES children, urine samples were collected and analyzed for 3,5,6-trichloropyridinol (TCPy), the primary urinary metabolite for chlorpyrifos (Nolan et al. 1984). First-morning-void urine samples were collected from the CPPAES children on each of the sampling days −1 (preapplication), 1, 2, 3, 5, 7, 9, and 11. These urine samples were designed to represent the contact of the children with chlorpyrifos on days −1, 0, 1, 2, 4, 6, 8, and 10, respectively, and estimate body burden, although there would be higher uncertainty in these values because it was a first void and not a 24-hr average (Wessels et al. 2003). The preapplication urine sample was collected as a baseline urine measure for TCPy concentration. Only 10% of the urine samples collected were not the first morning voids, and only two children missed more than one morning void (H5 and H9). The urine samples were analyzed by the Centers for Disease Control and Prevention. The samples were analyzed for TCPy using a slightly modified version of the method described by Hill et al. (1995) using a 3-hr derivatization process. The analytical limit of detection (LOD) for TCPy concentration using this method was 1.0 μg/L for a 4-mL sample. Results for both creatinine (CR) adjusted (micrograms TCPy/grams CR) and non-CR-adjusted (micrograms TCPy/liters urine) TCPy concentrations are reported in this study. The TCPy levels for 5 of the 80 urine samples were reported as less than the analytical LOD. We assumed a value of 0.5 × LOD for these samples, which is a generally accepted method of reporting data below the LOD (U.S. EPA 1999).

Translating the non-CR-adjusted morning void TCPy concentrations (micrograms TCPy/liters urine) to estimated daily TCPy excretion (micrograms per kilogram per day) required an assumption of 0.5 L/day daily urinary excretion rate for children between 0 and 4 years of age (Lentner 1981). The CR-adjusted first-morning-void TCPy concentrations (micrograms TCPy/grams CR) required an assumption of 25 mg CR/kg/day excretion rate (Hay et al. 1997) to estimate daily excretion of TCPy (micrograms per kilogram per day). However, there are uncertainties associated with both estimates. Based on the daily TCPy excreted amounts, the daily estimated amounts of chlorpyrifos absorbed by each of the CPPAES children via all routes were calculated using the approach of Byrne et al. (1998):


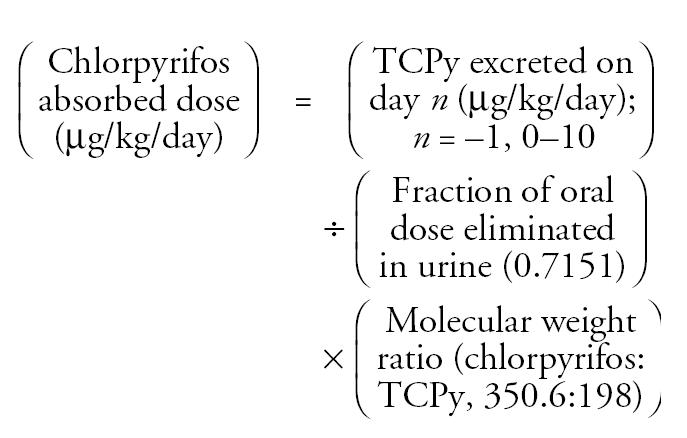


### Chemical analysis.

We used a capillary gas chromatograph (Hewlett-Packard 5860 Series II; all equipment from Hewlett-Packard, Wilmington, DE) equipped with an HP Nickel 63 electron capture detector and an Autosampler Injector 7673 for chlorpyrifos analysis of the air, surface wipe, and toy samples. We used HP Chem Station chromatography software to quantify the concentration of chlorpyrifos in all of the samples. A split/splitless injector was maintained at 250°C. The detector temperature was held at 325°C. A 60-m (0.25 mm inner diameter DB-1) fused silica capillary column with 0.25 μm film thickness (J&W Scientific, Folsom, CA) was used. Under splitless conditions, the column was temperature programmed from 50°C to 190°C at 30°C/min (held for 28 min), and from 190°C to 270 °C at 70°C/min, and held at 270°C for 16 min, altogether resulting in a run time of approximately 50 min per sample run. Helium was used as the carrier gas (flow rate, 1.0 mL/min). Nitrogen was used as the makeup gas (flow rate, 65 mL/min). An injection volume of 1 μL was maintained for all of the samples.

### Instrument quality assurance and quality control.

Standard solutions for chlorpyrifos ranged from 0.0012 to 2.4 μg/mL. These were analyzed with every gas chromatograph run, and calibration curves were generated for the concentration range of interest. The results were used to generate a linear regression equation (*r* ^2^ = 0.99). Replicates of independent standard solutions (prepared by Chem Service, Linden, NJ) were included with each sample run to evaluate the performance of the gas chromatograph. Pesticide recoveries from the independent standards (*n* = 10) were within 2% of the reported values, with CVs < 2.1%. All solvent blanks remained free of chlorpyrifos. Where no peaks were detected, the sample results were reported as nondetects (ND). The instrument LOD for chlorpyrifos was 0.0011 μg/mL.

### Statistical analyses.

CPPAES was designed specifically to study the mechanisms of release and exposure to semivolatile pesticides over a 2-week period postapplication. Thus, the emphasis of the study was on the time course of accumulation and elimination of a pesticide in a variety of media in the same home. Thus, it was not a population-based study. Because three of the homes received approximately five orders of magnitude lower amounts of chlorpyrifos, the CPPAES homes were divided into two groups based on application rate: “high” (H1–H7) and “low” (H8–H10). We used the Wilcoxon signed ranks test to compare the pretreatment and the 2-week posttreatment chlorpyrifos levels as measured from the indoor environment (air, dust, plush toys) and from the children (chlorpyrifos absorbed dose) within these groups. Given the mechanistic design of the study, there was a small sample size, and a nonparametric analysis method was employed to examine between group data. Using the nonparametric Mann-Whitney *U*-test, we compared the extent of daily average postapplication chlorpyrifos levels between the “high” and the “low” homes. This type of study was previously recommended as part of a modeling workshop (ILSI 2000).

## Results

### Indoor air samples.

**Figure 1:**
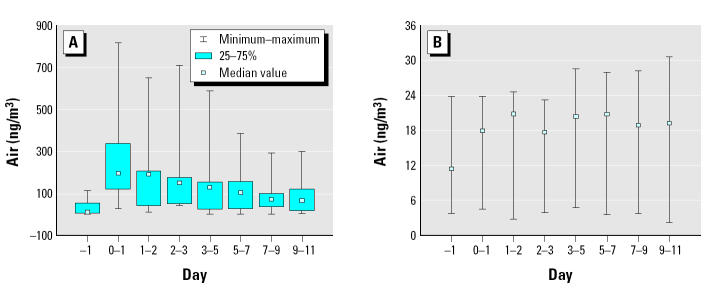
Box plots for chlorpyrifos concentrations in indoor air (ng/m^3^) for (*A*) “high” homes (H1–H7) and (*B*) “low” homes (H8–H10). Note that the y-axis on each plot is not the same.

Based on estimated chlorpyrifos application rates for homes H1–H7 (> 4.3 × 10^−6^ g) and homes H8–H10 (4.1 × 10^−7^ to 4.3 × 10^−6^ g) (Table 2), air concentrations of chlorpyrifos were categorized into two groups designated “high” and the “low” homes, respectively. Box plots of the indoor air chlorpyrifos concentrations measured throughout the 2-week period are presented in Figure 1.

### Surface wipes.

**Figure 2:**
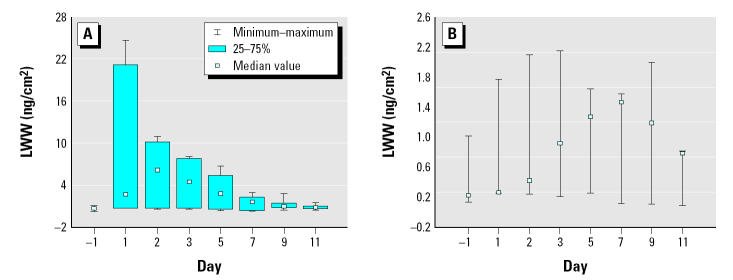
Box plots for chlorpyrifos surface loadings (main play areas, LWWA) (ng/cm^2^) for (*A*) “high” homes (H1–H7) and (*B*) “low” homes (H8–H10). Note that the y-axis on each plot is not the same.

**Figure 3:**
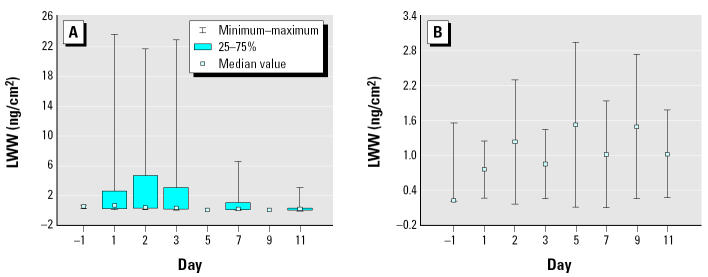
Box plots for chlorpyrifos surface loadings (bedroom areas, LWWB) (ng/cm^2^) for (*A*) “high” homes (H1–H7) and (*B*) “low” homes (H8–H10). Note that the y-axis on each plot is not the same.

The LWW wipe sample results obtained from the samples collected in the main play areas (A) and bedroom areas (B) are found in Tables 3 and 4, respectively. Box plots of the chlorpyrifos surface loadings for the 2-week period are presented in Figures 2 and 3, respectively.

Chlorpyrifos levels in the main play areas of the “high” homes (H3–H6) were considerably greater than the levels measured in homes H8–H10. For days 0–10, the average ranged from 3.1 to 6.9 ng/cm^2^ (H3–H6) and from 0.17 to 1.7 ng/cm^2^ (H8–H10). Despite the lower chlorpyrifos application rates in H8–H10, chlorpyrifos levels were detected; in fact, H10 chlorpyrifos levels (days 0–10 mean = 1.7 ng/cm^2^) were higher than levels measured in homes H1, H2, and H7 (days 0–10, mean range = 0.4–1.0 ng/cm^2^). The preapplication level in H10 (1.0 ng/cm^2^) suggested another source contributed to H10 chlorpyrifos levels. A potential source could be previous pesticide applications made within the home. LWW area A chlorpyrifos surface loadings in H3–H6 peaked between days 1 and 2 postapplication (mean = 13 ng/cm^2^), and the levels were significantly greater than the preapplication levels (< 0.9 ng/cm^2^; *p* = 0.006). After the peak day, the loading gradually declined approaching pretreatment levels by day 11 (mean = 0.9 ng/cm^2^). Surface loadings in homes H8 and H10 did not follow the same decay pattern. The highest loading postapplication for H8 (1.6 ng/cm^2^) was observed on day 7, and for H10 (2.1 ng/cm^2^) on day 3. Postapplication surface loadings in homes H8–H10 (mean = 0.93 ng/cm^2^) were only slightly greater than preapplication levels (mean = 0.44 ng/cm^2^). Levels reached or approached pretreatment levels on day 11 (mean = 0.55 ng/cm^2^).

Chlorpyrifos levels measured in the bedroom areas were generally lower than levels measured in the main play areas for H1–H7, excluding H5. In fact, except for H3 and H5, the highest postapplication surface loadings measured in the bedroom areas were only slightly greater than the preapplication levels (range = 0.18–0.82 ng/cm^2^; pretreatment = 0.28–0.70 ng/cm^2^). The highest LWW area A and area B surface loadings were measured in H5, with loadings peaking on day 1 postapplication (range = 21.2–23.8 ng/cm^2^; pretreatment levels were ND). After the peak day, loadings in H5 gradually declined approaching pretreatment levels on day 11.

### Plush toys.

**Figure 4:**
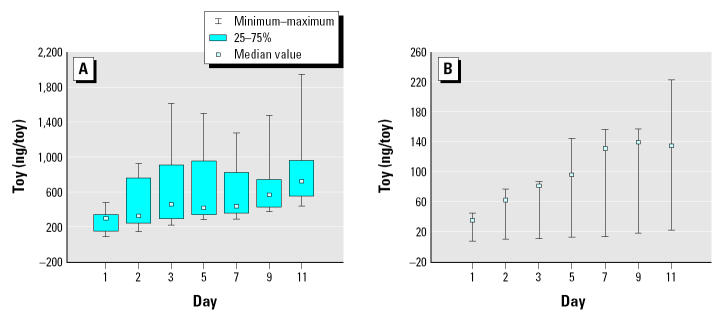
Box plots for chlorpyrifos concentrations within reference plush toys (ng/toy) for (*A*) “high” homes (H1–H7) and (*B*) “low” homes (H8–H10). Note that the y-axis on each plot is not the same.

Chlorpyrifos levels found in/on the plush toys are presented in Table 5 and illustrated in Figure 4. Chlorpyrifos concentrations in/on the plush toys increased throughout the 2-week sampling period for all homes. A similar trend was observed by Gurunathan et al. (1998), after a broadcast application of chlorpyrifos. On day 1, the plush toy chlorpyrifos concentrations for CPPAES homes H1–H10 averaged 197 ng/toy, reaching 634 ng/toy on day 11. Overall, levels measured within homes H1–H7 were significantly higher than levels in homes H8–H10 (*p* = 0.000). Measured chlorpyrifos levels were the highest in H5 throughout the 2-week period. Less than 5% (mean ± SD = 1.6 ± 2.0%; *n* = 26) of the chlorpyrifos was wiped off the plush toys (mean ± SD = 3.4 ± 2.6 ng). These amounts were significantly less than the amounts of chlorpyrifos obtained from the toys after full extraction (mean ± SD = 519 ± 606 ng; *p* = 0.000).

### Biomonitoring.

**Figure 5:**
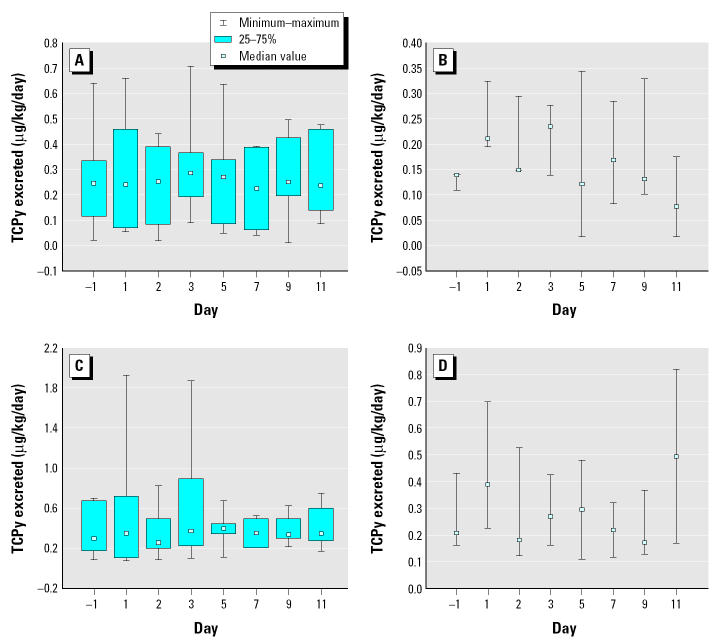
Box plots for daily TCPy excreted amounts measured from the CPPAES children postapplication (H1–H7 vs. H8–H10) (μg TCPy/kg/day). Non-CR-adjusted (*A*) “high” homes (H1–H7) and (*B*) “low” homes (H8–H10); CR-adjusted (*C*) “high” homes (H1–H7) and (*D*) “low” homes (H8–H10).

**Figure 6:**
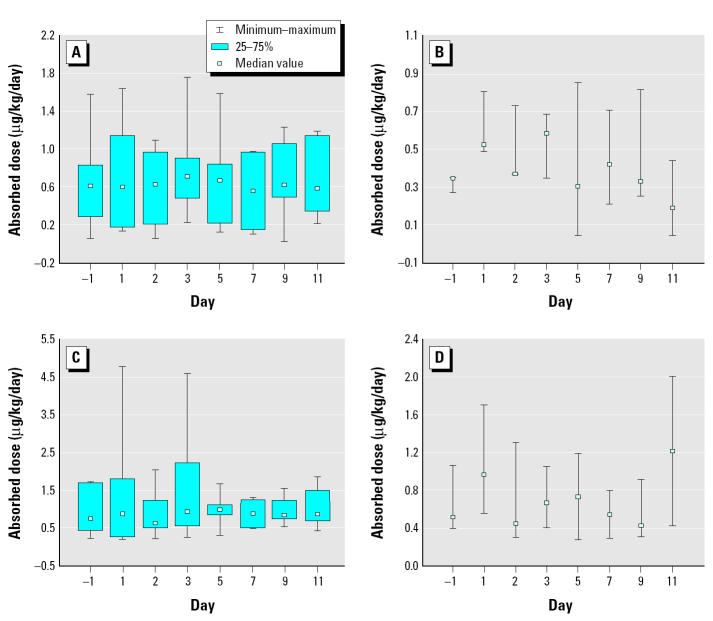
Box plots for daily chlorpyrifos absorbed doses calculated for the CPPAES children postapplication (H1–H7 vs. H8–H10) (μg chlorpyrifos/kg/day). Non-CR-adjusted (*A*) “high” homes (H1–H7) and (*B*) “low” homes (H8–H10); CR-adjusted (*C*) “high” homes (H1–H7) and (*D*) “low” homes (H8–H10).

We estimated chlorpyrifos levels absorbed by the CPPAES children by quantifying the amount of chlorpyrifos metabolite TCPy that was excreted by the children on the sampled days. The amount of TCPy excreted by the CPPAES children and the corresponding absorbed doses derived from both the non-CR-adjusted and the CR-adjusted TCPy results are presented in Table 6 and illustrated in Figures 5 and 6, respectively. However, CR is at lower levels in children, and there is probably a higher level of variability due to the lack of a 24-hr sample. The CPPAES children excreted on average approximately 0.25 μg TCPy/kg/day (non-CR adjusted; *n* = 10) or 0.34 μg TCPy/kg/day (CR adjusted; *n* = 10) preapplication. The estimated average chlorpyrifos absorbed doses were 0.55 μg chlorpyrifos/kg/day (non-CR adjusted) and 0.85 μg chlorpyrifos/kg/day (CR adjusted). The amount of TCPy excreted by the children postapplication on average per day ranged from 0.21 to 0.28 μg TCPy/kg/day (non-CR adjusted) and from 0.31 to 0.51 μg TCPy/kg/day (CR adjusted). The corresponding daily average postapplication chlorpyrifos absorbed doses ranged from 0.53 to 0.7 μg chlorpyrifos/kg/day (non-CR adjusted) and from 0.77 to 1.3 μg chlorpyrifos/kg/day (CR adjusted). A significant increase was not observed in the amount of chlorpyrifos absorbed by the CPPAES children during the 2-week period after the crack-and-crevice application.

## Discussion

CPPAES combined extensive multimedia monitoring efforts within residential homes for a 2-week period after a crack-and-crevice application of chlorpyrifos with simultaneous biomonitoring of the children residing within the treated homes. Biomonitoring of the chlorpyrifos metabolite enabled us to quantify the extent of aggregate exposure to the pesticide for a child living within a treated residence and estimate the body burden levels. Although previous studies have examined the time-series distribution of chlorpyrifos within an indoor environment, no studies thus far have concurrently measured the time-series urine levels from children that lived within the pesticide-treated homes and spent most of their time indoors. Moreover, because three of the homes (H8–H10) received approximately five orders of magnitude lower amounts of the chlorpyrifos, the reduced level of application gave us an opportunity to investigate the distribution of the pesticide within the home and the children after different application rates.

Some of the findings from CPPAES were in agreement with other studies that have demonstrated that semivolatile pesticides applied indoors within a home can contaminate the indoor air (Byrne et al. 1998; Gurunathan et al. 1998; Lewis et al. 2001; Wright and Leidy 1978, 1980; Wright et al. 1981) and nontargeted indoor surfaces (Gurunathan et al. 1998; Wright 1976; Wright and Jackson 1975).

Chlorpyrifos applied inside the 10 CPPAES homes was detected within the treated room indoor air throughout the 2-week postapplication period. Mostly, higher pesticide levels were detected from the CPPAES homes that received a greater application rate (except H7). For homes H1–H6, 2-week postapplication indoor air levels ranged from 22 to 816 ng/m^3^; H8–H10 levels ranged from 2.2 to 31 ng/m^3^. Comparatively, overall CPPAES concentrations in the indoor air were either similar or considerably lower than some of the reported studies. For instance, a study conducted by Wright and Leidy (1978) measured chlorpyrifos concentrations in the air within vacant rooms after a crack-and-crevice application of 0.5 or 1% chlorpyrifos solution. Pesticide measurements were made from the indoor air throughout a 3-day period after a crack-and-crevice application. Chlorpyrifos levels in the indoor air as measured immediately after the indoor application ranged from 600 to 2,700 ng/m^3^. A more recent study was conducted by Byrne et al. (1998) to estimate chlorpyrifos levels within pesticide-treated homes for a 10-day period after a crack-and-crevice application made with a 0.5% pesticide solution. The study was conducted in three residential homes. An estimated 3.3–3.9 g of chlorpyrifos was applied to each of the homes. Preapplication indoor air levels from the CPPAES homes were more or less comparable with measurements collected by Byrne et al. (1998) from two of the three treated homes (< 20 ng/m^3^). The highest indoor air chlorpyrifos level measured postapplication in the CPPAES study (816 ng/m^3^), however, was lower than the maximum concentration (2,300 ng/m^3^) observed by Byrne et al. (1998).

As a measure of the extent of nontarget deposition of the chlorpyrifos within the CPPAES homes after the crack-and-crevice application, postapplication surface loading measurements were made from nontreated surfaces within the treated homes. The highest postapplication chlorpyrifos loadings, as measured via wipe sampling from nontargeted surfaces within the CPPAES children’s main play areas and main living areas, were observed within homes H1–H7 (range = 0.03–24.6 ng/cm^2^). However, not all of the measured postapplication loadings from homes H1–H7 were higher than the corresponding levels from homes H8–H10 (range = 0.08–3 ng/cm^2^). Higher measured loadings in the children’s main play areas were not always accompanied with higher loadings in the children’s main living areas (except H5). Factors such as cleaning of the homes and tracking in or out of home soil/dust most likely contributed to the 2-week distribution of the indoor measured surface loadings. The levels observed on the indoor surfaces in the CPPAES were similar but somewhat higher than levels observed in the Minnesota Children’s Pesticide/National Human Exposure Assessment Survey study (median = 0.34 and 0.42 ng/cm^2^ for two different rooms in each home; maximum = 3.64 and 14.4 ng/cm^2^ for the same rooms) (Lioy et al. 2000). The latter were obtained in homes that used pesticides such as chlorpyrifos but were not necessarily measured immediately postapplication.

Other studies have reported similar or lower indoor levels of chlorpyrifos after crack-and-crevice treatments. In a study conducted by Wright and Jackson (1975), chlorpyrifos measurements were made from nontargeted surfaces (aluminum pie plates) placed within vacant dormitory rooms for an 8-day period after indoor crack-and-crevice pesticide applications with either 0.5 or 1% chlorpyrifos solutions. Chlorpyrifos deposition levels measured from the 0.5 or 1% treated areas ranged from 0.4 to 3.5 ng/cm^2^ and 0.4 to 11.3 ng/cm^2^, respectively, with overall pesticide levels decreasing throughout the 8-day period. The higher measured nontargeted surface loadings found in the present study, compared with levels measured in the reported studies with a greater application rate, may have resulted from a number of reasons. For instance, although the intention of this study was to sample from nontargeted surfaces, some of the nontargeted surfaces may have accidentally been applied with chlorpyrifos. Some of the variability observed in the surface concentrations may have resulted from the different sampling techniques that were used between the studies. Moreover, less activity within the treated rooms, such as walking or children playing, particularly in the dormitory study conducted by Wright and Jackson (1975), may have contributed to lower pesticide loadings on the nontargeted surfaces because of less redistribution and resuspension of the indoor dust.

In this study, we also examined pesticide levels on nontreated surfaces such as plush toys because children living within pesticide-treated homes may come into contact with contaminated objects, such as toys, within a home environment (Gurunathan et al. 1998). Moreover, similar sorbant surfaces such as furniture upholstery can also contain pesticides that children residing within treated homes can be exposed to. Chlorpyrifos concentrations measured from the plush toys that were placed within homes H1–H7 were significantly greater than levels measured from toys placed within homes H8–H10. H1–H7 chlorpyrifos levels ranged from 87 to 1,949 ng/toy; H8–H10 levels ranged from 7 to 221 ng/toy. Chlorpyrifos concentrations in/on the CPPAES plush toys increased throughout the 2-week sampling period, demonstrating a cumulative trend.

An increase in chlorpyrifos levels within the CPPAES homes provided an opportunity for increased exposure postapplication. However, although an increase was observed in the amount of chlorpyrifos measured from the CPPAES homes after the crack-and-crevice application, a significant increase was not observed in the amount of chlorpyrifos absorbed by the CPPAES children during the 2-week period after the crack-and-crevice application (Figures 5 and 6). Moreover, even though chlorpyrifos levels as measured from the various media within the indoor environment were considerably greater in the “high” homes compared with the “low” homes (indoor air ~ 10-fold; indoor surfaces ~ 4-fold; plush toys ~ 8-fold), postapplication daily absorbed chlorpyrifos doses measured from the “high” home children were only slightly greater (~ 2-fold) than levels measured from the “low” home children, essentially indicating that the children in fact were not coming into contact with all of the chlorpyrifos within the indoor environment, and the body burden levels could have been due to multiple sources, a point previously described by Krieger et al. (2003). The children’s activities may in fact have played an important role in determining how much pesticide each child actually absorbed. Total absorbed doses of chlorpyrifos as estimated for the children residing within the CPPAES treated homes (< 4.8 μg/kg/day) were within a factor of 2.5 of the chlorpyrifos doses estimated by Byrne et al. (1998) (< 2.1 μg/kg/day). The potential absorbed doses for children residing within three chlorpyrifos-treated homes were calculated by Byrne et al. (1998) using environmental data gathered after a crack-and-crevice application. The estimated body burden levels, however, could not be compared with the environmental results because body burden levels were not measured for children by Byrne et al. (1998).

Most (~ 97%) of the postapplication CPPAES children’s estimated absorbed doses (range = 0.02–4.8 μg/kg/day) were lower than the U.S. EPA oral reference dose (RfD) value of 3 μg/kg/day [based upon a no observed effect level of 30 μg/kg/day; calculated without the additional 10× safety factor added by FQPA (1996) to protect young children]. However, most (88%) of the 10 CPPAES children’s estimated absorbed doses exceeded the revised RfD value of 0.3 μg/kg/day (calculated including the additional 10× safety factor) by up to 1,600%. The EPA in their final risk assessment for chlorpyrifos had considered a safety factor of 3 as opposed to a more conservative FQPA safety factor of 10, which reduced the number of estimated exceedances. Only 29% of the CPPAES children’s estimated absorbed doses exceeded the RfD of 1 μg/kg/day (calculated using the safety factor of 3).

Comparison of results from CPPAES and Gurunathan et al. (1998) suggests that selection of the application method will greatly influence the children’s exposures and dose received from pesticides applied indoors. In particular, comparison of the results of these two studies has indicated that estimated pesticide body burden levels for children living within homes after a broadcast application of a semivolatile pesticide were considerably greater than the measured body burden levels for the children living within crack-and-crevice–treated homes. For instance, the total absorbed doses of chlorpyrifos for children residing within the crack-and-crevice–treated homes were considerably lower than even the nondietary estimated doses from Gurunathan et al. (1998) (208–356 μg/kg/day). One possible reason may have been that the pesticide levels in the indoor environment on child-accessible objects after the broadcast application by Gurunathan et al. (1998) were considerably greater than the levels measured in the crack-and-crevice studies. This was not unexpected because, compared with the Gurunathan et al. (1998) broadcast application, the CPPAES crack-and-crevice application method required a smaller volume of the pesticide applied. For instance, whereas approximately 296–473 mL of chlorpyrifos formulation containing ~ 0.07–1.8 g of chlorpyrifos was applied to the CPPAES homes, approximately 2,000 mL of a chlorpyrifos formulation yielding 12 g of chlorpyrifos was applied to surfaces in each Gurunathan et al. (1998) apartment. The highest indoor air chlorpyrifos level measured postapplication in the study by Gurunathan et al. (1998) was 7,000 ng/m^3^. Whereas cumulative pesticide concentrations measured from the CPPAES toys were < 1,949 ng/toy, plush toy cumulative pesticide concentrations measured by Gurunathan et al. (1998) reached levels > 30,000 ng/toy. Consequently, residents of a crack-and-crevice–treated home would be potentially exposed to lower amounts of the pesticide.

Some data gaps introduce uncertainties during interpretation of CPPAES postapplication urinary TCPy results. For instance, there is limited information available on the natural variability in background urinary TCPy levels, a point that needs to be kept in mind because the values are relatively low. Moreover, both CR-adjusted and non-CR-adjusted TCPy data have inherent limitations. For example, no studies have systematically evaluated the validity of using CR adjustment for children. Moreover, the accuracy of TCPy values derived from samples with CR levels ≤30 mg/dL urine is questioned by Lauwerys and Hoet (1993) as being too dilute to provide valid results.

## Conclusions

CPPAES results indicate that when chlorpyrifos is applied properly via a crack-and-crevice mode of application, the application does not lead to a significant increase in the children’s chlorpyrifos body burden levels. Although an increase was observed in the amount of chlorpyrifos measured from the CPPAES homes after the pesticide application, CPPAES findings indicated that the children living within the crack-and-crevice–treated homes were actually not coming into contact with most of the chlorpyrifos that was present in the indoor environment. Thus, pesticide body burden levels estimated for children living within crack-and-crevice–treated homes, which are considerably lower than levels estimated for children living within homes treated via broadcast application (Gurunathan et al. 1998), had other sources. Essentially, adjusting the mode of application so as to spray the pesticide around pest-infested targeted areas rather than an entire surface area of a house greatly reduced the amount of pesticide that children living within treated homes would potentially be exposed to and uptake after an application.

## Figures and Tables

**Table 1:** Sample collection scheme for the CPPAES homes.

	Preapplication	Day of application	*n*th day postapplication							
Day	−1	0	1	2	3	4	5	7	9	11

**Table 2:** Indoor air measurements for chlorpyrifos within treated rooms (ng/m^3^).

	Days								
Home identification	^−^1–0	0–1	1–2	2–3	3–5	5–7	7–9	9–11	Average (days 0–10)
“High” homes									
H1	3.4	179	195	178	132	123	87	73	138
H2	10	121	130	71	29	31	39	22	63
H3	7.2	338	207	153	155	107	73	69	157
H4	58	312	203	164	145	158	102	122	172
H5	14	816	648	709	587	386	294	299	534
H6	115	196	44	55	41	45	46	50	68
H7	18	32	14	45	5.5	4.0	4.4	6.3	16
Average	32	285	206	196	156	122	92	92	—
Median	14	196	195	153	132	107	73	69	—
SD	41	257	210	233	199	129	95	99	—
“Low” homes									
H8	3.8	4.5	2.8	4.0	4.8	3.6	3.7	2.2	3.7
H9	12	18	21	18	20	21	19	19	19
H10	24	24	25	23	29	28	28	31	27
Average	13	15	16	15	18	17	17	17	—
Median	12	18	21	18	20	21	19	19	—
SD	10	9.9	12	9.9	12	13	12	14	—

**Table 3:** Surface loading measurements for chlorpyrifos from nontargeted surfaces within treated rooms (main play areas, LWWA) (ng/cm^2^).

	Day								
Home identification	−1	1	2	3	5	7	9	11	Average (days 0–10)
“High” homes									
H1	ND	1.89	1.03	1.02	NA	0.71	NA	0.49	1.03
H2	0.10	0.49	0.60	0.59	0.24	0.18	0.29	0.33	0.39
H3	ND	2.55	6.04	4.39	1.96	2.81	2.69	1.36	3.11
H4	ND	24.6	10.9	4.48	3.40	1.46	0.75	0.61	6.60
H5	ND	21.2	10.1	7.93	5.26	1.71	1.33	0.83	6.90
H6	0.85	16.5	9.6	7.7	6.6	2.2	0.82	0.83	6.32
H7	0.57	0.46	0.42	0.45	0.46	0.25	0.67	0.67	0.48
Average	0.51	9.7	5.5	3.8	3.0	1.3	1.1	0.73	—
Median	0.57	2.6	6.0	4.4	2.7	1.5	0.79	0.67	—
SD	0.38	10.7	4.8	3.2	2.6	1.0	0.85	0.33	—
“Low” homes									
H8	0.21	0.24	0.41	0.91	1.3	1.6	1.2	0.81	0.91
H9	0.12	0.25	0.23	0.19	0.24	0.10	0.09	0.08	0.17
H10	1.0	1.8	2.1	2.1	1.6	1.5	2.0	0.8	1.70
Average	0.44	0.75	0.91	1.08	1.05	1.04	1.09	0.55	—
Median	0.21	0.25	0.41	0.91	1.26	1.46	1.18	0.78	—
SD	0.49	0.88	1.00	0.99	0.72	0.82	0.96	0.41	—

NA, not available.

**Table 4:** Surface loading measurements for chlorpyrifos from nontargeted surfaces within treated rooms (bedroom areas, LWWB) (ng/cm^2^).

	Day								
Home identification	−1	1	2	3	5	7	9	11	Average (days 0–10)
“High” homes									
H1	ND	0.18	0.10	0.12	NA	0.18	NA	0.16	0.15
H2	0.63	NA	0.27	0.18	0.10	0.05	0.07	0.16	0.14
H3	ND	2.7	4.7	3.1	NA	1.1	NA	0.03	2.3
H4	0.28	0.29	0.47	0.26	NA	0.20	NA	0.30	0.30
H5	ND	23.8	21.8	23.0	NA	6.6	NA	3.1	15.7
H6	0.49	0.82	0.41	0.40	NA	0.34	NA	0.23	0.44
H7	0.70	0.49	0.41	0.38	NA	0.15	NA	0.27	0.34
Average	0.53	4.7	4.0	3.9	0.10	1.20	0.07	0.61	—
Median	0.56	0.66	0.41	0.38	0.10	0.20	0.07	0.23	—
SD	0.19	9.4	8.0	8.5	—	2.4	—	1.1	—
“Low” homes									
H8	0.21	NA	NA	NA	NA	NA	NA	NA	—
H9	0.23	0.27	0.17	0.26	0.11	0.10	0.26	0.28	0.21
H10	1.6	1.3	2.3	1.5	3.0	1.9	2.8	1.8	2.1
Average	0.67	0.76	1.24	0.86	1.54	1.02	1.50	1.03	—
Median	0.23	0.76	1.24	0.86	1.54	1.02	1.50	1.03	—
SD	0.78	0.70	1.5	0.84	2.0	1.3	1.76	1.1	—

NA, not available.

**Table 5:** Chlorpyrifos levels in/on reference plush toys placed within treated rooms (ng/toy).

	Day						
Home identification	1	2	3	5	7	9	11
“High” homes							
H1	329	761	911	957	578	665	721
H2	189	278	342	343	362	427	442
H3	344	445	672	625	824	746	753
H4	150	247	300	420	374	374	962
H5	481	926	1,615	1,495	1,275	1,480	1,949
H6	302	328	457	384	434	566	588
H7	87	145	221	284	293	437	552
Average	269	447	646	644	592	671	852
Median	302	328	457	420	434	566	721
SD	135	289	490	440	350	382	512
“Low” homes							
H8	7.3	10	11	13	13	18	22
H9	45	62	81	96	130	139	134
H10	35	76	87	144	156	157	221
Average	29	50	60	84	100	105	126
Median	35	62	81	96	130	139	134
SD	19	35	42	66	76	75	100

**Table 6:** Amount of TCPy excreted in urine calculated for the CPPAES children (μg/kg/day).

	Home Identification									
Day	H1	H2	H3	H4	H5	H6	H7	H8	H9	H10
Non-CR-adjusted										
−1	0.22	NA*^a^*	0.11	0.02*^a^*	0.34*^b^*	0.64	0.28	0.14	0.11	0.14*^a^*
	(0.53)	(NA)*^a^*	(0.28)	(0.04)*^a^*	(0.83)*^b^*	(1.6)	(0.68)	(0.35)	(0.27)	(0.35)*^a^*
1	0.14	0.29	0.05	0.07	0.24^a,b^	0.46	0.66	0.21	0.20*^a^*	0.33
	(0.35)	(0.71)	(0.14)	(0.18)	(0.60)^a,b^	(1.1)	(1.6)	(0.53)	(0.49)*^a^*	(0.81)
2	0.22	0.44	0.08	0.02^a,c^	0.26	0.39	0.25	0.15	0.30	0.15
	(0.53)	(1.1)	(0.21)	(0.05)^a,c^	(0.64)	(0.97)	(0.63)	(0.37)	(0.73)	(0.37)
3	0.22	0.37	0.09	0.19	0.29*^b^*	0.71	0.32*^a^*	0.14	0.28	0.24
	(0.53)	(0.91)	(0.22)	(0.48)	(0.71)*^b^*	(1.8)	(0.78)*^a^*	(0.35)	(0.69)	(0.58)
5	0.24	0.34	0.05	0.27	0.27	0.64	0.086*^a^*	0.12	0.35	0.02*^c^*
	(0.58)	(0.84)	(0.12)	(0.67)	(0.68)	(1.6)	(0.21)*^a^*	(0.30)	(0.86)	(0.04)*^c^*
7	0.39	0.28	0.04*^a^*	0.06*^a^*	0.21	0.39	0.22	0.17	0.29	0.08
	(0.97)	(0.70)	(0.10)*^a^*	(0.15)*^a^*	(0.52)	(0.97)	(0.56)	(0.42)	(0.71)	(0.21)
9	0.20	0.25	0.01*^c^*	0.24	0.43	0.50	0.28	0.33	0.13*^b^*	0.10
	(0.49)	(0.62)	(0.02)*^c^*	(0.58)	(1.1)	(1.2)	(0.69)	(0.82)	(0.33)*^b^*	(0.25)
11	0.15*^a^*	0.48	0.14	0.24	0.34	0.46	0.086*^a^*	0.18*^a^*	0.08*^b^*	0.02*^c^*
	(0.38)*^a^*	(1.2)	(0.34)	(0.58)	(0.83)	(1.1)	(0.21)*^a^*	(0.44)*^a^*	(0.19)*^b^*	(0.04)*^c^*
CR-adjusted										
−1	0.30	NA*^a^*	0.09	NA*^a^*	0.68*^b^*	0.70	0.18	0.21	0.16	0.43*^a^*
	(0.74)	(NA)*^a^*	(0.22)	(NA)*^a^*	(1.7)*^b^*	(1.7)	(0.44)	(0.51)	(0.40)	(1.1)*^a^*
1	0.38	0.35	0.08	0.10	1.9^a,b^	0.73	0.26	0.23	0.70*^a^*	0.39
	(0.93)	(0.87)	(0.19)	(0.25)	(4.8)^a,b^	(1.8)	(0.64)	(0.56)	(1.7)*^a^*	(0.96)
2	0.30	0.20	0.09	NA*^a^*	0.50	0.83	0.21	0.18	0.53	0.12
	(0.74)	(0.50)	(0.22)	(NA)*^a^*	(1.2)	(2.0)	(0.52)	(0.45)	(1.3)	(0.30)
3	0.23	0.38	0.10	0.30	0.40*^b^*	0.90	1.9*^a^*	0.16	0.27	0.42
	(0.56)	(0.93)	(0.25)	(0.74)	(1.0)*^b^*	(2.2)	(4.6)*^a^*	(0.40)	(0.67)	(1.0)
5	0.45	0.40	0.12	0.38	0.43	0.68	0.35*^a^*	0.11	0.48	NA
	(1.1)	(0.99)	(0.29)	(0.93)	(1.1)	(1.7)	(0.86)*^a^*	(0.27)	(1.2)	(NA)
7	0.38	0.21	NA	0.21*^a^*	0.50	0.53	0.33	0.32	0.22	0.12
	(0.93)	(0.51)	(NA)	(0.51)*^a^*	(1.2)	(1.3)	(0.82)	(0.79)	(0.54)	(0.29)
9	0.63	0.35	NA	0.22	0.30	0.50	0.33	0.37	0.13*^b^*	0.17
	(1.5)	(0.87)	(NA)	(0.53)	(0.74)	(1.2)	(0.82)	(0.91)	(0.31)*^b^*	(0.42)
11	0.60*^a^*	0.35	0.17	0.38	0.28	0.75	0.32*^a^*	0.82*^a^*	0.17*^b^*	NA
	(1.5)*^a^*	(0.87)	(0.43)	(0.93)	(0.68)	(1.9)	(0.79)*^a^*	(2.0)*^a^*	(0.42)*^b^*	(NA)

NA, not available. Chlorpyrifos-absorbed doses within parentheses.

a Sample dilute: urine samples with CR levels < 30 mg/dL urine (Lauwerys and Hoet 1993).

b Not morning void urine sample.

c Analyte (TCPy) concentrations were < 1 μg/L (LOD for a 4-mL sample). For these a value of 0.5 × LOD (i.e., 0.5 μg/L) was assumed. Daily total urine volume excretion was assumed to be 0.5 L (Lentner 1981); CPPAES children’s body weights H1–H10 = 25, 14, 25, 14, 16, 14, 14, 18, 15, and 14 kg, respectively. Daily CR excretion rate was assumed to be 25 mg of CR/kg/day (average of the 20–30 mg of CR/day excretion rate for children suggested by Hay et al. (1997). Chlorpyrifos absorbed doses were calculated using the equation presented in “Materials and Methods.”
